# De Novo Transcriptome Assembly of *Anoectochilus roxburghii* for Morphological Diversity Assessment and Potential Marker Development

**DOI:** 10.3390/plants13233262

**Published:** 2024-11-21

**Authors:** Wenting Zhang, Ke Chen, Yu Mei, Jihua Wang

**Affiliations:** 1 Crop Research Institute, Guangdong Academy of Agriculture Sciences, Guangzhou 510640, China; zhangwenting@gdaas.cn (W.Z.); meiyu@gdaas.cn (Y.M.); 2 Guangdong Provincial Key Laboratory of Crops Genetics and Improvement Guangdong, Guangdong Academy of Agriculture Sciences, Guangzhou 510640, China; 3 Guangdong Provincial Engineering & Technology Research Center for Conservation and Utilization of the Genuine Southern Medicinal Resources, Guangdong Academy of Agriculture Sciences, Guangzhou 510640, China; 4 Rice Research Institute, Guangdong Academy of Agricultural Sciences, Guangzhou 510640, China; chenke@gdaas.cn; 5 Guangdong Rice Engineering Laboratory, Guangdong Academy of Agricultural Sciences, Guangzhou 510640, China; 6 Key Laboratory of Genetic and Breeding of High Quality Rice in Southern China (Co-Construction by Ministry and Province), Ministry of Agricultural and Rural Affairs, Guangdong Academy of Agricultural Sciences, Guangzhou 510640, China

**Keywords:** *Anoectochilus roxburghii*, transcriptome assembly, cultivars, diversity, SSR markers

## Abstract

*Anoectochilus roxburghii* is a rare and precious medicinal and ornamental plant of Orchidaceae. Abundant morphological characteristics have been observed among cultivated accessions. Our understanding of the genetic basis of morphological diversity is limited due to a lack of sequence data and candidate genes. In this study, a high-quality de novo transcriptome assembly of *A.roxburghii* was generated. A total of 138,385 unigenes were obtained, and a BUSCO (Benchmarking Universal Single-Copy Orthologs) analysis showed an assembly completeness of 98.8%. Multiple databases were used to obtain a comprehensive annotation, and the unigenes were functionally categorized using the GO (Gene Ontology), KOG (Eukaryotic Orthologous Groups), KEGG (Kyoto Encyclopedia of Genes and Genomes), and Nr databases. After comparing the phenotypic characteristics of five representative cultivars, a set of cultivar-specific, highly expressed unigenes was identified based on a comparative transcriptome analysis. Then, a WGCNA (Weighted Gene Co-expression Network Analysis) was performed to generate gene regulatory modules related to chlorophyll content (red) and sucrose synthase activity (black). In addition, the expression of six and four GO enrichment genes in the red and black modules, respectively, was analyzed using qRT-PCR to determine their putative functional roles in the leaves of the five cultivars. Finally, in silico SSR (Simple Sequence Repeat) mining of the assembled transcriptome identified 44,045 SSRs. Mononucleotide was the most dominant class of SSRs, followed by complex SSRs. In summary, this study reports on the phenomic and genomic resources of *A. roxburghii*, combining SSR marker development and validation. This report aids in morphological diversity assessments of *Anoectochilus roxburghii*.

## 1. Introduction

*Anoectochilus roxburghii* (Wall.) Lindl., a perennial herb of Orchidaceae, typically has attractive, velvety leaves that are often patterned with silver or metallic green veins. The distinctive foliage is one of the reasons for its common name, “jewel orchid”. The species is native to regions of East Asia, including China, India, Japan, Laos, Myanmar, Thailand, and Vietnam. In addition to its aesthetic appeal, *A. roxburghii* has cultural significance in traditional Chinese medicine. Extracts from the plant are traditionally used in Chinese herbal medicine for their potential health benefits [1,2]. It is believed to have antioxidant and anti-inflammatory properties due to its high polysaccharides content [3,4]. Efforts to identify the exact bioactive fractions and decipher their mechanisms of action will help uncover their potential benefits for human health.

In addition to its medicinal use, *A. roxburghii* is also cultivated for its ornamental value. The sizes, colors, and vein patterns of the leaves of *A. roxburghii* from different provenances are significantly different [5,6]. The swift assembly of novel genomes and transcriptomes is uncovering the fundamental molecular genetic mechanisms of leaf development, yielding a profound comprehension of leaf diversity and its evolutionary aspects [7]. Whether through conventional or molecular approaches, the genetics improvement of *A. roxburghii* has faced challenges due to restricted genomic resources and limited genetic diversity within the elite gene pool. All these call for genomic research aiming to address challenges in its genetic improvement due to limited resources and diversity.

Illumina paired-end sequencing is a high-throughput method that allows sequencing both ends of DNA fragments, providing more information than single-end sequencing. This approach improves the accuracy of assembly and helps in identifying structural variations and duplications. The advent of next-generation sequencing (NGS) technologies has facilitated the generation of extensive genomic resources, encompassing transcriptome sequence data, molecular markers, and genetic and physical maps. These advancements facilitate more precise trait mapping and enable efficient marker-assisted breeding, allowing researchers and breeders to identify and select for desirable traits with greater accuracy. This, in turn, supports the development of improved crop varieties that are better suited to meet agricultural challenges and consumer needs. To date, no complete genome sequence of *A. roxburghii* has been reported, which restricts the development of functional genomics and modern breeding. According to a previous karyotype analysis, *A. roxburghii* is a perennial homozygous tetraploid (2n = 4x = 80) crop with the 2C value of DNA being approximately 6.83 ± 0.067 pg [8,9,10]. This implies that sequencing the entire genome would incur significant costs. Therefore, utilizing a transcriptome analysis for gene annotation is considered the preferred approach, highlighting the need for extensive studies on the *A. roxburghii* transcriptome.

RNA sequencing (RNA-seq) stands as a powerful tool to unravel the intricacies of the transcriptome, proving particularly advantageous for species lacking a sequenced genome. The abundance of obtained reads can be assembled to annotate genes, make gene discoveries, analyze gene expression, and identify regulatory patterns within organisms. Furthermore, RNA-seq has proven to be instrumental in pinpointing potential molecular markers, streamlining trait mapping, and enabling marker-assisted selection, even in the absence of a reference genome. This technology has found widespread application in transcriptome analyses across diverse species [11,12,13]. WGCNA is a statistical method used to analyze the relationships between genes based on their expression levels. Researchers often use it to identify clusters of co-expressed genes, find modules associated with traits, and explore the functional significance of gene networks. Few *A. roxburghii* transcriptome analyses have been reported for morphological diversity, most are about response to stress, like heat [14,15], light [16], cadmium [17], mycorrhizal fungus [18,19], etc. Currently, only one study is available to reveal how pigment biosynthesis influence chlorina leaf formation in *A. roxburghii* [5]. By identifying gene modules associated with specific leaf traits, we can delve into the genetic basis of morphological variations and uncover potential regulatory pathways. We therefore aimed to explore how significant variations in leaf traits are established across different cultivars based on WGCNA.

Simple sequence repeat (SSR) molecular markers are preferred markers for molecular genetic research in crops due to their co-dominant, multi-allelic nature; abundance in the genome; ease of scoring; and, most importantly, their PCR-based characteristics. In silico mining of simple sequence repeats (SSRs) from assembled transcriptomes is an efficient and cost-effective method for SSR development. This technique leverages bioinformatics tools to identify and characterize SSRs in transcriptomic data without the need for extensive laboratory work. In silico approaches allow for the rapid scanning of large transcriptome datasets, enabling the identification of numerous SSRs in a single analysis [20]. By using existing transcriptomic data, researchers can minimize costs associated with sequencing and library preparation. This approach is particularly beneficial for non-model organisms where genomic resources may be limited [21,22]. SSRs identified from transcriptomes are often associated with expressed genes, making them potentially more relevant for functional studies and genetic mapping [23,24,25]. This relevance enhances their utility in breeding programs and genetic diversity studies. SSRs mined in silico can be quickly validated through PCR amplification, which allows for efficient marker development for various applications, including population genetics and phylogenetic studies [23,25]. To date, SSR mining of *A. roxburghii* has not been reported. Thus, our goal was to generate a reference RNA sequence for *A. roxburghii* that can function as a molecular toolbox.

In view of this, the present study involved the de novo assembly of transcriptomes for four tissues (root, stem, stem node, and leaf) of JXL28, a representative local cultivar of *A. roxburghii* from Luofu Mountain in Guangdong, using the Illumina paired-end sequencing strategy. We also compared the morphological and transcriptomic features among five different *A. roxburghii* genotypes, focusing on plant height, leaf color, leaf size, and leaf vein pattern. Through transcriptome-based Weighted Gene Co-expression Network Analysis (WGCNA), we identified genes associated with chlorophyll content and sucrose synthase activity. To further explore SSR locus information and develop molecular markers, we examined unigenes from the JXL28 transcriptome. The efficacy of the developed SSR markers was validated by assessing genetic diversity across 20 accessions. Overall, our findings contribute significantly to the field of *A. roxburghii* research by enhancing our understanding of the genetic basis underlying important agronomic traits. The development of molecular markers will facilitate more effective breeding strategies, ultimately supporting the conservation and sustainable utilization of this valuable but endangered herb. By linking transcriptomic data to phenotypic traits, this study provides essential resources for future research and breeding programs, paving the way for improved cultivation practices and enhanced medicinal properties in *A. roxburghii*.

## 2. Materials and Methods

### 2.1. Sample Collection

All five *A. roxburghii* accessions (JXL10, JXL14, JXL15, JXL21, and JXL28) conserved at the Crop Research Institute, Guangdong Academy of Agriculture Sciences, Guangzhou, China were used for a transcriptome analysis based on their leaf traits and polysaccharide content. As a rare medicinal herb with very slow growth, *A. roxburghii* takes approximately six months to develop five leaves. To facilitate sample collection and ensure consistency, we performed stable tissue culture for all samples and set six months as the time standard for subsequent sampling. As a representative local cultivar from Luofu Mountain in Guangdong, JXL28 was used for de novo assembly, and its root, stem, and leaf tissues were sampled separately for transcriptome sequencing. As to the other four accessions, the whole six-month-old seedlings were used for transcriptome sequencing. Utilizing different accessions of *A. roxburghii* enhances the robustness of the transcriptome analysis by capturing genetic diversity and potential variations in leaf traits and polysaccharide content, thereby providing comprehensive insights into the species’ functional genomics and improving the understanding of its medicinal properties.

### 2.2. Sequencing Library Construction and Sequencing

Oligo(dT)-attached magnetic beads were used to purify mRNA in three replicates by eliminating rRNA and tRNA in a total amount of 2 μg RNA per sample. The purified mRNA was fragmented into small pieces with a fragment buffer at an appropriate temperature. Then, first-strand cDNA was generated via random hexamer-primed reverse transcription, followed by a second-strand cDNA synthesis. After that, RNA Index Adapters and A-Tailing Mix were added by incubating to end repair. The cDNA fragments obtained from the previous steps were amplified using PCR, and the PCR products were purified using Ampure XP Beads (Agencourt, Beverly, MA, USA) and dissolved in an EB solution. The products were validated on an Agilent Technologies (Santa Clara, CA, USA) 2100 bio-analyzer for quality control. The double-stranded PCR products obtained from the previous steps were denatured via heating and circularized via the splint oligo sequence to obtain the final library. The final constructed libraries were further amplified with phi29 to make DNA nanoballs (DNBs). The DNBs were loaded into a patterned nano-array, and paired ends of 150 bp base reads were generated on a T7 platform by Wuhan Benagen Technology Co., Ltd. (Wuhan, China). The raw data were processed with FastQC (http://www.bioinformatics.babraham.ac.uk/projects/fastqc/ (accessed on 22 July 2022)) to filter out adapters and low-quality sequences. This detailed protocol highlights the critical steps in constructing a high-quality RNA sequencing library, essential for generating reliable and reproducible transcriptomic data.

### 2.3. Transcriptome Assembly and Functional Annotation

The assembly of high-quality clean reads was performed using Trinity software (v2.13.2 --normalize_max_read_cov 50 --min_kmer_cov 5 --min_glue 10). The trinity-assembled transcript sequences were concatenated, and hierarchical clustering was performed using Corset (version 1.09) [26] with the default parameters. The longest cluster sequence obtained from the clustering was identified as the unigene for a subsequent analysis.

Multiple databases (KEGG, NR, Swiss-Prot, GO, and COG/KOG) were used for functional annotation of the transcripts. Clustered transcripts were annotated using the homology approach—to assign functional annotation—and the BLAST tool (Diamond v2.0.9 -e 1e-5 --max-hsps 1 --more-sensitive -k 1) [27], and amino acid sequences were aligned with the Pfam database to predict possible functions using HMMER software (v3.3.2), with an *E*-value of 10^−10^ as the threshold. Further, a Gene Ontology (GO) analysis was carried out using ClusterProfiler (v4.6.0) [28] to assign the contigs to the three GO terms: cellular component, molecular function, and biological process. This comprehensive approach to assembling and annotating transcript sequences not only ensures the generation of high-quality unigenes but also provides critical functional insights through extensive database comparisons, facilitating a deeper understanding of gene roles in *A. roxburghii*.

### 2.4. Gene Expression Analysis

The unigene abundance was normalized using reads per kilobase of exon model per million mapped reads (FPKMs). Unigenes were defined as unique or shared expressed transcripts among contrasting tissues based on the FPKM value (FPKM > 0). A differential expression analysis of two groups (JXL28 was assigned as the control and others as treated) was performed using the DESeq2 R package (1.10.1) [29]. The resulting *p* values were adjusted using Benjamini and Hochberg’s approach for controlling the false discovery rate (FDR). Genes with a threshold of FDR ≤ 0.05 and an absolute value of log_2_^Ratio^ ≥ 1 were assigned as differentially expressed.

### 2.5. Weighted Gene Co-Expression Network Analysis

Gene co-expression matrices were created using the R software (v4.1.2) package “WGCNA” [30]. After discarding undetectable or relatively low expression genes, the co-expression modules were obtained using the automatic network construction function (blockwiseModules) with default parameters, apart from at a soft threshold power of 18, where TOMtype was signed. According to the criteria of the hybrid dynamic shear tree, the minimum number of genes in each gene network module was set to 40, the characteristic genes of each module were calculated, and the modules were clustered with the height set to 0.4. This rigorous application of WGCNA in constructing gene co-expression matrices is crucial for identifying biologically relevant modules, facilitating the exploration of gene interactions and their associations with specific traits in *A. roxburghii*.

### 2.6. Validation of RNA Seq Data via qRT-PCR

Primers were designed with the help of NCBI Primer-BLAST (https://www.ncbi.nlm.nih.gov/tools/primer-blast/index.cgi?LINK_LOC=BlastHome (accessed on 2 July 2023)). Total RNA was extracted from each sample using a SteadyPure Plant RNA Extraction Kit (Accurate Biotechnology (Changsha, Hunan, China), cat#AG21019) according to the manufacturer’s instructions. Each sample (2 μg) of total RNA was used to synthesize the first-strand cDNA with the HiScript III All-in-one RT SuperMix Perfect for qPCR (Vazyme (Nanjing, China), cat#R333-01). RT-qPCR was performed using a CFX96 Touch Real-Time PCR Detection System (Bio-Rad, Hercules, CA, USA) with the Taq Pro Universal SYBR qPCR Master Mix (Takara, Japan, Cat# RR820A). The expression levels of target genes were normalized against those of *A. roxburghii* ACTIN. Error bars represent the SD from three independent experiments. The relative fold change in the selected genes was calculated using the 2^−ΔΔCt^ algorithm. This comprehensive methodology for qRT-PCR ensures accurate quantification of gene expression in *A. roxburghii*, enabling reliable comparisons of target gene activity while standardizing against ACTIN, which is essential for understanding the functional roles of these genes.

### 2.7. SSRs Mining and Characterization

Unigenes greater than 1000 bps were screened for microsatellite or simple sequence repeats (SSRs) using MISA software (https://webblast.ipk-gatersleben.de/misa/misa_sourcecode_25082020.zip (accessed on 25 November 2022)) with the recommended default parameters. If the distance between two SSRs is less than 100 bp, they form a compound SSR.

Additionally, SSR primer pairs were designed using Primer 3 (v2.5.0) software. A total of 25 SSR primer pairs were randomly selected, synthesized, and used for the validation of the PCR amplification and polymorphism detection in 20 *A. roxburghii* cultivars with optimized annealing temperatures. PCR was performed in a 15 μL reaction mixture containing 7.5 μL PowerPol 2X PCR Mix with Dye (ABclonal, Wuhan, China), 20 pM of each of the forward and reverse primers, and 100 ng of genomic DNA in the ETC811 plus PCR system (Dongshenglong, Beijing, China), and volume makeup was conducted with sterilized distilled water. The thermal cycling program followed for the PCR amplifications of the SSRs was 95 °C for 3 min, 35 cycles of 95 °C for 40 s, 57–60 °C (annealing temperatures for different SSR primers) for 30 s and 72 °C for 1 min, and a final step at 72 °C for 8 min. The PCR products were separated on 3.5% agarose gels containing 0.1 mg/mL of ethidium bromide in a 1X TBE buffer via electrophoresis at 70–75 V for 3–4 h. A 2000 bp DNA ladder was run in each gel with PCR amplicons to estimate the amplicon sizes. The separated PCR bands in the gels were visualized by exposing the gel to UV light in the GelDoc Go system (Bio-Rad, Hercules, CA, USA), and gel images were captured. This meticulous approach to screening and validating SSRs in *A. roxburghii* not only aids in developing genetic markers for cultivar differentiation but also enhances the understanding of genetic diversity within the species, which is crucial for breeding and conservation efforts.

### 2.8. Extraction and Detection of Total Polysaccharides

We first homogenized the roots, stems, and leaves of 6-month-old and 12-month-old *A. roxburghii*. The water extracting–alcohol precipitating method is used to isolate polysaccharides from *A. roxburghii*. First, dried plant material was ground into a fine powder and soaked in hot water (about 80 °C) for several hours. The mixture is then filtered to separate the liquid extract from the solid residue. Ethanol (usually 70%) is added to the filtered extract, causing the polysaccharides to precipitate. After centrifugation to collect the precipitate, it is washed with ethanol and dried, yielding the extracted total polysaccharides for further analysis.

Following the phenol-sulfuric acid method [31], we mixed the extracts with 1 mL of 5% phenol solution and then quickly added 5 mL of concentrated sulfuric acid. After allowing the mixture to stand at room temperature for 10–30 min to observe color changes, we measured the absorbance at 490 nm using a spectrophotometer. Quantification was performed using a standard curve with known polysaccharide concentrations, enabling us to compare total polysaccharide levels across different plant parts and ages.

### 2.9. Detection of Sugar by GC-MS Analysis

Sugar contents were detected by MetWare (http://www.metware.cn/ (accessed on 7 July 2022)) based on the Agilent 8890-5977B platform. Agilent 8890 (Agilent technologies, Santa Clara, CA, USA) gas chromatograph coupled to a 5977B mass spectrometer with a DB-5MS column (30 m length × 0.25 mm i.d. × 0.25 µm film thickness) was employed for GC-MS analysis of sugars. Helium was used as carrier gas at a flow rate of 1 mL/min. Injections were made in the split mode with a split ratio 5:1 and the injection volume was 1 μL. The oven temperature was held at 170 °C for 2 min, and then raised to 240 °C at 10 °C/min, raised to 280 °C at 5 °C/min, raised to 310 °C at 25 °C/min and held at the temperature for 4 min. All samples were analyzed in selective ion monitoring mode. The ion source and transfer line temperatures were 230 °C and 240 °C, respectively.

### 2.10. Morphological and Physiological Data Collection

Agronomic characteristics, including weight, height, leaf number, diameter, aerial root number, stem node number, leaf length, and leaf width, were collected from 6-month-old plants of *A. roxburghii*. The Plant Chlorophyll Content Assay Kit (Cat.No.BC0990) and the Sucrose Synthetase (SS) Activity Assay Kit (Cat.No.BC0585) were purchased from Solarbio (Beijing, China) to detect the content of chlorophyll and the activity of sucrose synthetase.

## 3. Results

### 3.1. Transcriptome Assembly and Annotation

**Figure 1:**
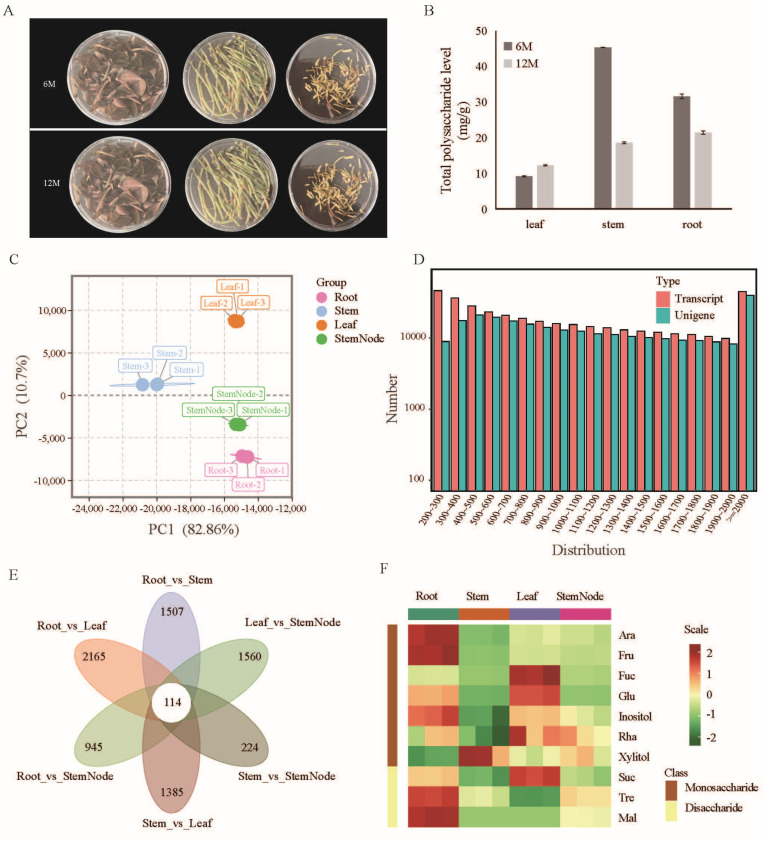
Transcriptome sequencing and sugar composition analysis of *A. roxburghii* (JXL28). (**A**) The leaf, stem, and root of JXL28, aged six (up) and twelve (down) months, respectively, are utilized for the assessment of total polysaccharide levels. (**B**) The total polysaccharide levels in the leaf, stem, and root of *A. roxburghii* after six and twelve months of growth. (**C**) A PCA analysis of various tissues of JXL28 transcriptome. (**D**) Length distribution of assembled transcripts and unigenes of the JXL28 transcriptome. (**E**) A Venn diagram showing differentially expressed unigenes unique to or shared among differential groups. (**F**) Heatmap of the levels of ten sugars in various tissues of JXL28. Ara: *D*-Arabinose; Fru: *D*-Fructose; Fuc: *L*-Fucose; Glu: Glucose; Mal: Maltose; Rha: *L*-Rhamnose; Suc: Sucrose; Tre: Trehalose.

The availability of genomic sequence resources is limited in *A. roxburghii*. Here, we report the de novo assembly and analysis of different tissue transcriptomes of *A. roxburghii* for the first time. At the very beginning, we first compared the differences in the total polysaccharide levels in the roots, stems, and leaves of 6- and 12-month-old *A. roxburghii*. We found that the total polysaccharide levels in the 6-month-old stems were the highest (Figure 1A,B). The polysaccharide extracted from *A. roxburghii* is a biologically active ingredient, with immunomodulatory, antioxidant, and anti-inflammatory bioactivities and pharmacological effects [32]. Previous studies have shown that the roots of *A. roxburghii* accumulate the highest levels of polysaccharides. Given that *A. roxburghii* is an aerial root plant, with roots sprouting from its stem nodes, we were curious as to whether there is a difference in sugar accumulation between the stems and the stem nodes. Thus, a transcriptome database for *A. roxburghii* was constructed from the stem nodes, stems, leaves, and roots (Figure 1C). This comprehensive dataset allows us to better understand the genetic basis underlying the observed polysaccharide accumulation patterns.

About 697 million sequence reads (96.60% of clean reads) available after sequence preprocessing were used for the de novo assembly of the transcriptome (Supplementary Table S1). A total of 250,084 transcripts were identified from the de novo assembled transcriptome, while 138,385 unigenes were identified after hierarchical clustering using Corset [26] (Supplementary Table S2). The length of the unigenes in the final assembly ranged from 201 to 16,889 bp, with an average length of 1544 bp, and the N90 value of 693 bp was observed (Figure 1D). To assess the quality of our transcriptome assembly, we employed BUSCO, which evaluates the integrity of genome assemblies by comparing them to a set of universally conserved genes [33]. A completeness assessment of the transcriptome assembly using BUSCO showed a completeness of 98.8%, i.e., 252 core genes were detected out of the 255 core genes queried. Based on the RNA-seq data, a hierarchical cluster analysis was performed on the differentially expressed unigenes (DEGs) in each comparison combination (Supplementary Figure S1). Furthermore, the differential unigenes that were unique to or shared among differential groups were determined (Figure 1E). Additionally, the sugar levels in different tissues were examined using gas chromatography (GC)/mass spectrometry (MS) (Figure 1F, Supplementary Table S3). The findings revealed that the concentrations of various monosaccharides and disaccharides varied among the different tissues of *A. roxburghii*. Different sugar levels across tissues can help the plant efficiently manage energy and resources according to the environmental conditions and developmental stages.

**Figure 2:**
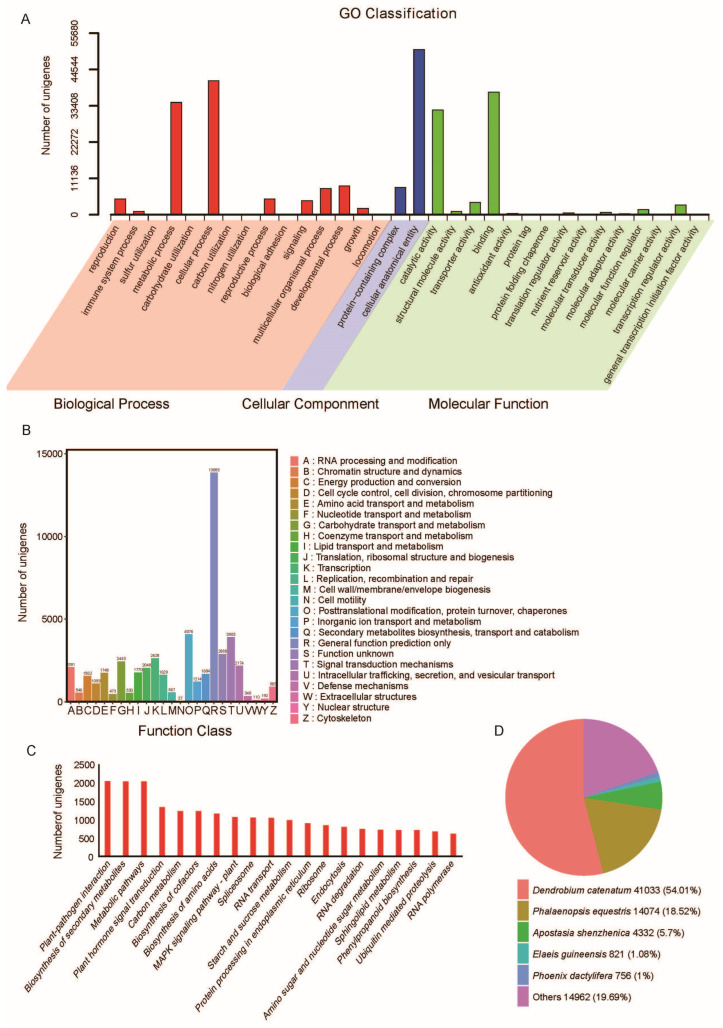
Functional annotation of unigenes. (**A**) GO ontology annotation of the *A. roxburghii* (JXL28) transcriptome showing the major GO terms in the molecular function, biological process, and cellular component categories. (**B**) Histogram representation of the cluster of orthologous group (COG) classification for assembled unigenes. (**C**) A KEGG analysis of the JXL28 transcriptome showing the top 20 highly represented KEGG pathways. The *X*-axis indicates the KEGG (Kyoto Encyclopedia of Genes and Genomes) pathways, and the *Y*-axis indicates the number of transcripts in each pathway. (**D**) Species-based distribution of blastx matches for each clustered unitranscript of the JXL28 transcriptome. The species with a match < 1% are grouped in the “Other” category.

The unigenes were functionally classified using Gene Ontology (GO), euKaryotic Ortholog Groups of proteins (KOG), and Kyoto Encyclopedia of Genes and Genomes (KEGG). A total of 64,184 unigenes were assigned to 46 GO terms, 45,620 unigenes were differentiated into 26 KOG categories, and 56,295 unigenes were assigned to 142 KEGG pathways (Figure 2, Supplementary Table S4). The Gene Ontology (GO) classification of the unigenes into the cellular process, molecular process, and biological process categories was performed to understand the transcriptome composition. In the molecular function category, “binding” (GO:0005488) and “catalytic activity” (GO:0003824) were the top two GO terms. In the biological process category, “cellular process” (GO:0009987) and “metabolic process” (GO:0008152) were the top two GO terms. In the cellular component category, “cellular anatomical entity” (GO:0110165) and “protein-containing complex” (GO:0032991) were the two GO terms (Figure 2A). The KOG assignment showed ten groups related to cellular processes and signaling (D, M, N, O, T, U, V, W, Y, and Z), eight groups related to metabolism (C, E, F, G, H, I, P, and Q), five groups related to information storage and processing (A, B, J, K, and L), and two poorly characterized groups (R and S) (Figure 2B). Among the 142 KEGG pathways, plant–pathogen interaction [PATH:ko04626] was the most abundant pathway in terms of the number of homologous unigenes, followed by the biosynthesis of secondary metabolites [PATH:ko01110] and metabolic pathways [PATH:ko01100] (Figure 2C). Among the BLASTx top hits, 41,033 (54.01%) were matched to *Dendrobium catenatum* proteins, followed by *Phalaenopsis equestris* (14,074; 18.52%), and *Apostasia shenzhenica* (4332; 5.7%) (Figure 2D).

### 3.2. A. roxburghii Morphological Diversity

**Figure 3:**
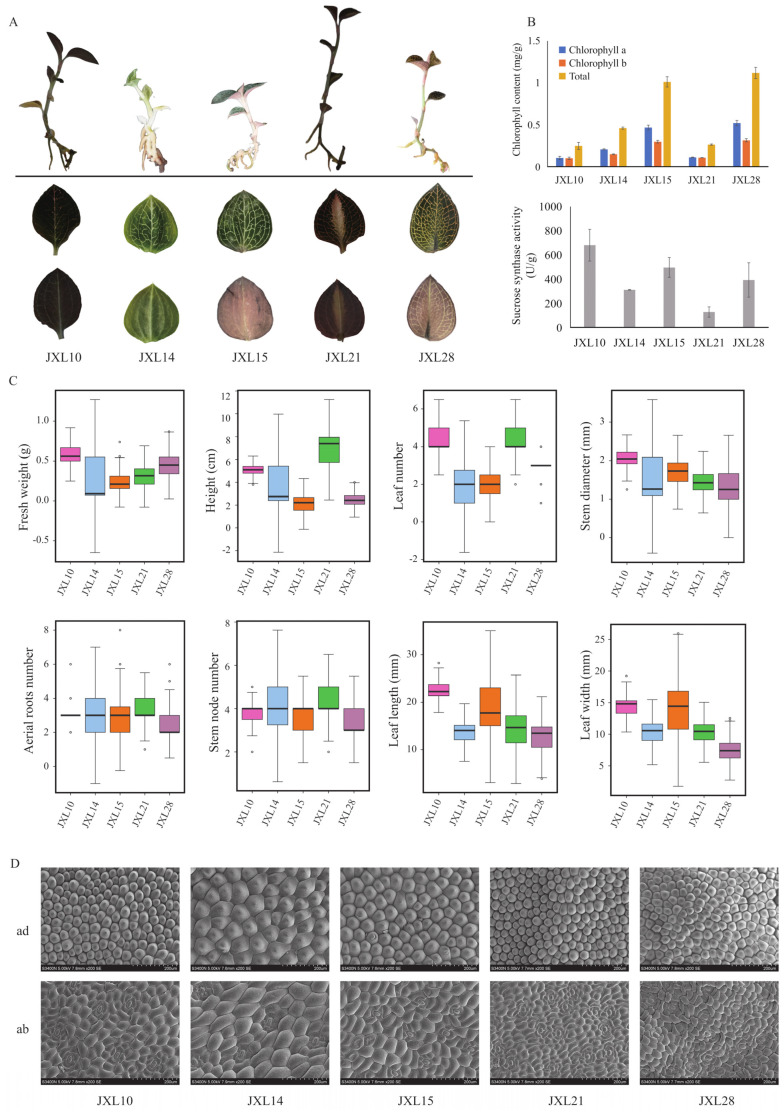
Morphological diversity of *A. roxburghii.* (**A**) Morphology of leaf adaxial, leaf abaxial, and seedling of five representative “Jinxianlian” cultivars. (**B**) An analysis of chlorophyll content (**up**) and sucrose synthase activity (**down**); analysis of five representative “Jinxianlian” cultivars. (**C**) Investigation of ten agronomic characteristics, including weight, height, leaf number, diameter, aerial root number, stem node number, leaf length, and leaf width. The hollow circles represent discrete values. (**D**) Comparison of leaf surface morphology among five representative “Jinxianlian” cultivars using scanning electron microscopy (SEM). ad: adaxial; ab: abaxial.

The morphological traits of *A. roxburghii* differ significantly among cultivars. We compared the morphological appearance of the 6-mouth-old seedlings and leaves of five representative cultivars (Figure 3A). The differences in plant height and leaf color are the most obvious. The leaves of JXL10 are dark green in both the adaxial and abaxial palisade, while those of JXL14 are light green. The golden color of the leaf veins is the most pronounced in JXL28, while in JXL14 and JXL15, the leaf veins are more whitish. In JXL10, the leaf veins are not noticeable due to the dark color of the leaves. Compared with the other three, the color difference between the adaxial and abaxial is clearer in JXL28 and JXL15. The discernible dissimilarity in appearance between JXL21 and JXL28 is that there is a light white stripe in the middle of the leaves of JXL21. These rich leaf shapes increase the aesthetic and commercial appeal of *A. roxburghii*.

Chlorophyll a (Chl a) and chlorophyll b (Chl b) are essential for photosynthesis, and their levels are key indicators of leaf greenness [34]. The levels of chlorophyll content were significantly higher in JXL15/JXL28 than in JXL10/JXL14/JXL21, which means that the dark green color mainly contributed the dark-colored leaves (Figure 3B, upper). The morphological diversity of *A. roxburghii* was also reflected in the leaf number, leaf length, leaf width, and stem diameter (Figure 3C). Additionally, JXL10 had the highest fresh weight, based on it having the highest stem diameter and largest leaf size. The number of aerial roots number and stem nodes did not differ significantly among these cultivars. In addition, leaf anatomical characteristics showed that the cells were the largest in JXL14, followed by JXL15, and the stomatal frequency was significantly higher in JXL21 and JXL28, while cells were longer and narrower in JXL21 (Figure 3D).

As an intermediate for the synthesis of many matrix polysaccharides, sucrose synthase (SuSy) is the key enzyme responsible for sucrose accumulation, while its activity is inconsistent in different species or different varieties of the same species. However, we did not find a significant correlation between SuSy activity and sucrose content. The SuSy activity was at least three times higher in JXL10 (the highest) than in JXL21 (the lowest) (Figure 3B, lower). As SuSy plays a crucial role in energy supply and carbon partitioning in plants, JXL10 may have advantages over JXL21 in terms of growth, development, or stress response. Higher SuSy activity could contribute to faster growth rates, better stress tolerance, or higher yield. This information could be useful for breeding programs. Varieties with higher SuSy activity, such as JXL10, may be preferred for certain agricultural or horticultural purposes due to their enhanced metabolic capabilities. The finding prompts further investigation into why there is such a disparity in SuSy activity between these varieties. It could involve genetic factors, environmental influences, or a combination of both. Understanding the underlying reasons could lead to more targeted approaches to crop improvement.

### 3.3. Transcriptomic Characterization of Five Representative Cultivars

**Figure 4:**
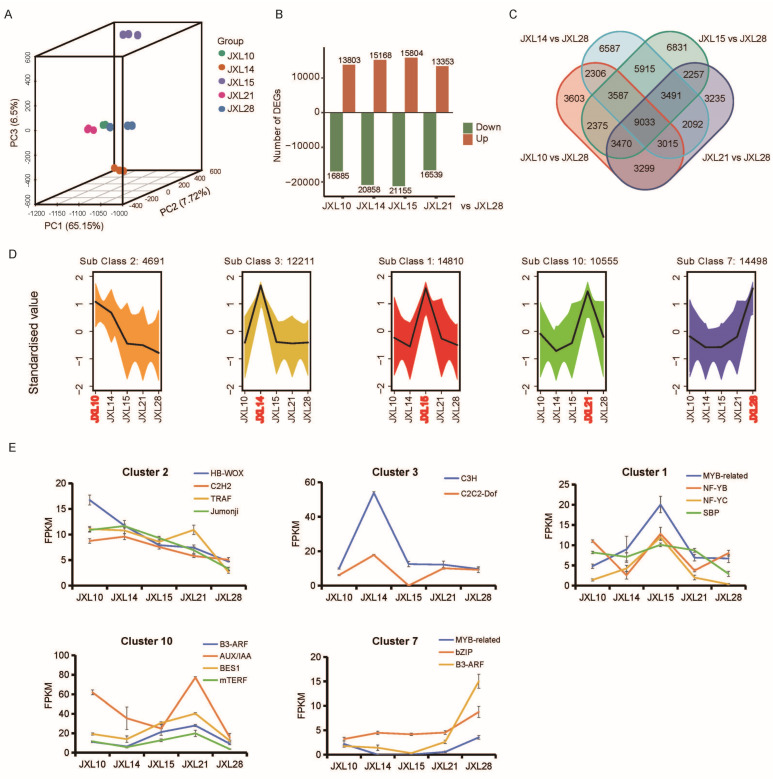
Transcriptome analysis of five representative *A. roxburghii* cultivars. (**A**) Results of 3D-PCA of five “Jinxianlian” cultivars based on the expression level of all unigenes, with each dot representing an independent experimental repeat. (**B**) Numbers of up- and down-regulated DEGs in each comparison (others vs. JXL28). (**C**) Venn diagram showing the number of DEGs in each combination. (**D**) Results of five main clusters from *K*-means clustering analysis. (**E**) RNA-seq results for several transcription factor candidate from five main clusters.

In order to identify putative genes related to morphological diversity in *Anoectochilus roxburghii*, we performed a comparative transcriptome analysis to investigate the variation in transcriptional regulation (Supplementary Figure S2). Among the five cultivars, JXL10 and JXL21 had a relatively high correlation (Supplementary Figure S3). As shown in Figure 4A, a three-dimensional principal component analysis (3D-PCA) was performed to evaluate the effect of cultivar diversity on unigene expression levels. Principal component 1 (PC1), representing 65.15% of the total variation, distinguished all five groups. Meanwhile, PC2 and PC3, representing 7.72% and 6.5% of the total variation, respectively, distinguished JXL14 and JXL15 from the others. After a comparison with the assembly transcriptome, the differentially expressed unigenes (DEGs) of each cultivar relative to JXL28 were identified and visualized in a volcano plot (Figure 4B, Supplementary Figure S4). A Venn diagram showed the distribution of DEGs between comparison groups (Figure 4C). A total of 9033 DEGs were contained in all comparisons.

To better investigate the effect of expression shifts on the morphological diversity among cultivars, we classified the unigenes into 10 subclasses based on their accumulation patterns using the *k*-means clustering algorithm [35] (Supplementary Figure S5). By analyzing the 10 subclasses, we found unigenes that were highly expressed in specific cultivars, such as Subclass 2 in JXL10, Subclass 3 in JXL14, Subclass 1 in JXL15, Subclass 10 in JXL21, and Subclass 7 in JXL28 (Figure 4D). To further associate the gene expression patterns with morphological characteristics, we performed a GO enrichment analysis to identify the functional unigenes that were correlated with morphogenesis (Supplementary Figure S6). The results showed the following: genes involved in cell division and cell wall biogenesis were enriched in JXL15; genes involved in phloem development were enriched in JXL10; genes involved in guard cell differentiation were enriched in JXL28; and genes involved in trichome morphogenesis were enriched in JXL21. Although multiple GO items were present in a single subclass, some clear patterns could be identified. For example, the most enriched GO item was “chalcone synthase activity” in Subclass 1, followed by “naringenin–chalcone synthase activity”, which means that the unigenes highly expressed in JXL15 are possibly related to the flavonoid biosynthetic process. FPKM values help compare the expression levels of genes within a sample and across different samples. We subsequently examined the FPKM levels of unigenes annotated as transcription factors in each subclass (Figure 4E) and whose expression pattern is consistent with results of the *k*-means analysis.

### 3.4. Identification of Candidate Biomarkers for Chlorophyll Content and Sucrose Synthase Activity Through a WGCNA

A weighted gene co-expression network analysis (WGCNA) can be used to identify highly correlated genes and group them into the same module. Additionally, these modules may be associated with specific external traits. As a statistical method for studying gene co-expression networks, WGCNA has demonstrated its advantages in screening hub genes and has been widely applied in many aspects [36,37].

**Figure 5:**
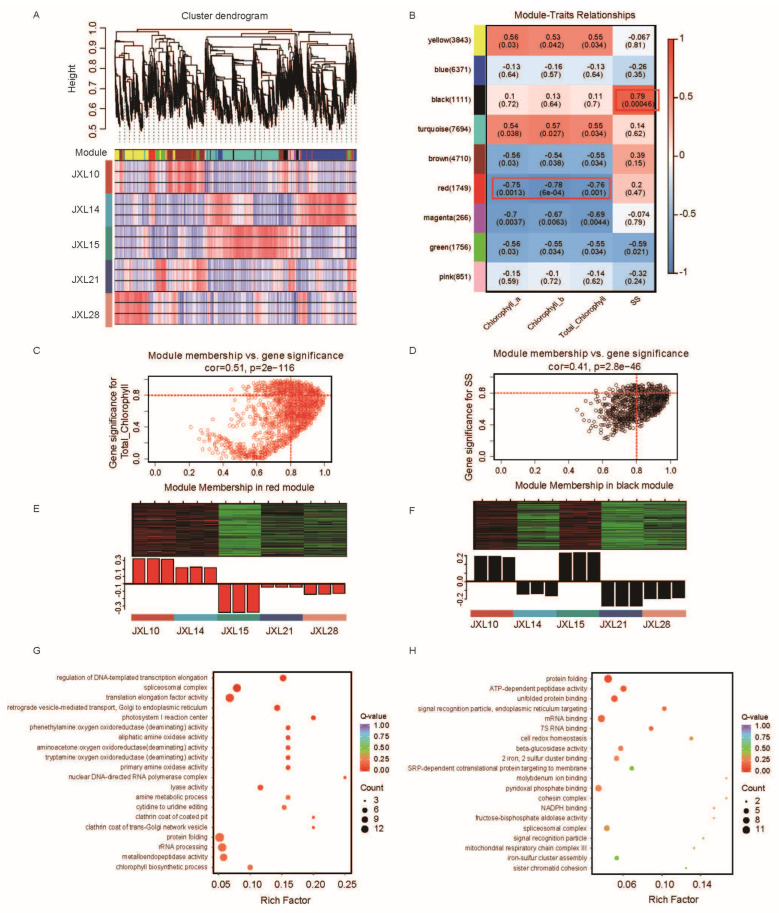
Identification of important modules and biomarkers based on a WGCNA. (**A**) A cluster dendrogram and the color display of co-expression network modules for all unigenes. (**B**) A correlation matrix of the module eigengene values obtained from the WGCNA. Nine modules were identified, and each module eigengene was tested for correlation with traits. In each cell, the upper values are the correlation coefficients between the module eigengenes and the traits; the lower values are the corresponding *p*-values; the co-expression modules significantly associated with the content of Chl a, Chl b, and total chlorophyll content and sucrose synthase activity are highlighted in red boxes. (**C**,**D**) A scatterplot describing the relationship between MM and GS in the red (**C**) and black (**D**) modules; key genes are screened out in the upper-right area, where GS > 0.8 and MM > 0.8. (**E**,**F**) A heatmap of the genes in the red (**E**) and black (**F**) modules; (**G**,**H**) A dotplot of the GO enrichment analysis of the genes in the red (**G**) and black (**H**) modules.

In our study, RNA-Seq data from these five cultivars were screened and filtered, and 28,352 valid genes were selected for a WGCNA. A hierarchical clustering tree was constructed based on the correlation of gene expression, with each branch representing a set of genes with highly correlated expressions (Figure 5A). In total, nine gene co-expression modules were identified. Each module is represented by a different color.

Building on this foundation, we conducted a correlation analysis between the nine gene co-expression modules and key traits, specifically chlorophyll content (Chl a and Chl b) and sucrose synthase activity (Figure 5B). The results showed that the co-expression modules significantly associated with the development of Chl a, Chl b, and total chlorophyll content were the same. Among them, the red module was significantly (*p* < 0.001) negatively correlated with chlorophyll content (r = −0.75, *p* = 0.0013; r = −0.78, *p* = 6 × 10^−4^; r = −0.76, *p* = 0.001), while the yellow module was slightly (*p* < 0.05) positively associated with chlorophyll content (r = 0.56, *p* = 0.03; r = 0.53, *p* = 0.042; r = 0.55, *p* = 0.034). Furthermore, the black module was positively correlated with sucrose synthase activity (r = 0.79, *p* = 0.00046).

To delve deeper into these relationships, we created a scatterplot to illustrate the association between module membership (MM) and gene significance (GS) for the red (Figure 5C) and black (Figure 5D) modules. Here, we identified key genes in the upper-right area, where GS > 0.8 and MM > 0.8 (Supplementary Figure S7). Meanwhile, Figure 5E,F provides a clear visual representation of the expression trends of the genes in these two modules across different samples. The upper part shows a clustering heatmap of the genes in the module, with red indicating high expression and green indicating low expression; the lower part displays the expression pattern of the module feature across different samples.

To further understand the biological implications of these findings, we performed GO and KEGG enrichment analysis based on genes in red and black modules (Figure 5G,H, Supplementary Figure S8). We focused on the chlorophyll biosynthetic process (GO:0015995) and beta-glucosidase activity (GO:0008422) from the GO results of the red and black modules, respectively. Here, genes encoding dicarboxylate diiron protein, NAD(P)-binding Rossmann-fold superfamily protein, and aldolase superfamily protein are related to the chlorophyll biosynthetic process, while genes encoding beta glucosidase 46 (BGLU46) and tetratricopeptide repeat (TPR)-like superfamily protein EMBRYO DEFECTIVE 2453 (EMB2453) are associated with the beta-glucosidase activity. Additionally, we noticed the circadian rhythm (ko04712) and the MAPK signaling pathway (ko04016) in the red and black modules based on the KEGG results. LHY (Late Elongated Hypocotyl), FT (Flowering Locus T), CO (CONSTANS), and ELF3 (Early Flowering 3) are key genes in the regulation of flowering time in plants, part of the photoperiod and circadian clock pathways. Here, LHY, FT, CO, and ELF3-like genes were identified in the circadian rhythm-plants item.

### 3.5. Assessment of Gene Expression by RT-qPCR

**Figure 6:**
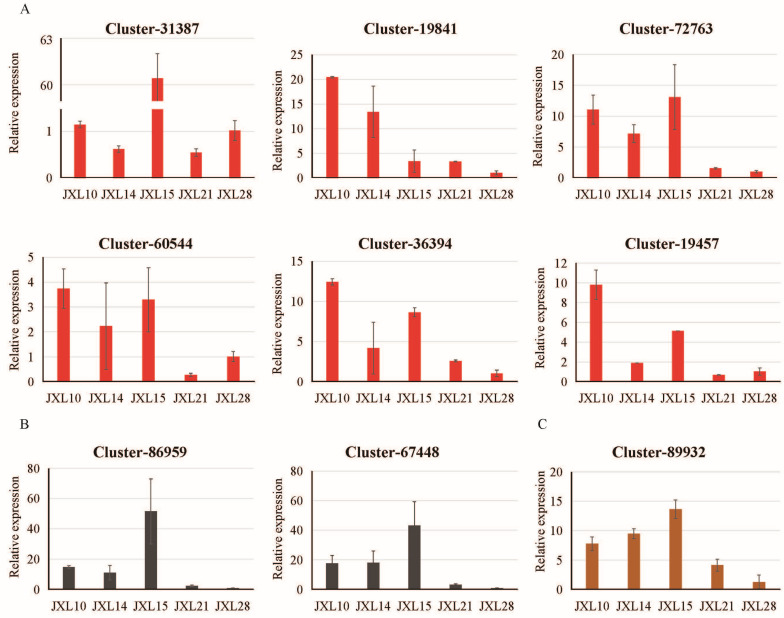
Verification of RNA-seq results via qRT-PCR of candidate unigenes. (**A**) Six unigenes selected from the hub gene of the red module. (**B**) Two unigenes selected from the hub gene of the black module. (**C**) One unigene selected from the DEGs. Error bars indicate SD (n = 3).

To validate the RNA-seq results, six and four genes encoding functional proteins identified in the red and black modules, respectively, were selected for qRT-PCR, including the low domain-containing transcription factor (Cluster-31387); transcription elongation factor family protein (Cluster-19841); photosystem I reaction center subunit IV (PsaE, Cluster-727630), which affects the effective quantum yield of photosystem II; FtsH extracellular protease (Cluster-60544); dicarboxylate diiron protein (Crd1, Cluster-36394); NAD(P)-binding Rossmann-fold superfamily protein (Cluster-19457); calreticulin 3 (CRT3, Cluster-86959), a member of the calcium-binding chaperones primarily localized in the endoplasmic reticulum; beta glucosidase 46 (BGLU46, Cluster-67448), which is involved in the lignin biosynthetic process; tetratricopeptide repeat (TPR)-like superfamily protein (Cluster-4552); and glutathione reductase, which is most likely localized in the chloroplast (Cluster-11174). We examined the expression of these genes both in leaf and stem of *Anoectochilus roxburghii*. The results showed that the expression levels in the stems were mostly lower than those in the leaves. However, Cluster-4552 and Cluster-11174 could not be detected in the leaves of JXL28 using RT-PCR. The expression profiles in the leaves of *A. roxburghii* for the other eight genes are shown in Figure 6A,B. The expression trends are basically consistent with the RNA-seq expression results, suggesting the accuracy and reliability of the transcriptome data. Additionally, a unigene (Cluster-89932, encoding duplicated homeodomain-like superfamily protein) differentially expressed between JXL28 and the other four cultivars was also examined via a qRT-PCR analysis (Figure 6C). The primers used for qRT-PCR are listed in Supplementary Table S5. Overall, these results enhance our understanding of the developmental regulatory networks governing the key traits of “Jinxianlian”, such as chlorophyll content and sucrose synthase activity, and they offer new information for future research on *Anoectochilus roxburghii*.

### 3.6. SSR Characterization and Cross-Cultivar Amplification

**Figure 7:**
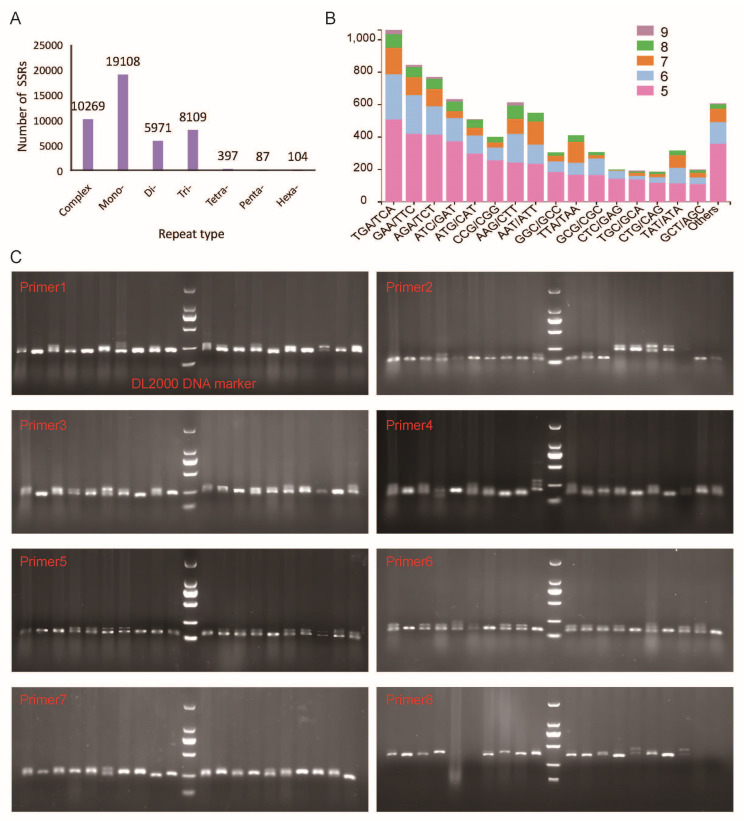
Characterization of potential simple sequence repeat (SSR) markers using MISA software. (**A**) The distribution of the different nucleotide repeat types (complex; Mono—mononucleotide; Di—dinucleotide; Tr—trinucleotide; Tetra—tetranucleotide; Penta—pentanucleotide; Hexa—hexanucleotide). (**B**) A stacked bar chart representing the abundance of trinucleotide repeats. (**C**) PCR amplification of genic-SSR markers in 20 *A. roxburghii* genotypes.

SSR markers are important tools for studying genetic diversity, constructing genetic maps, and performing comparative genomics. Potential SSR markers were detected from unigenes with by using MISA software. A total of 44,045 SSRs were mined from 34,946 unigenes, of which 7304 sequences contained more than one SSR, and 10,268 SSRs were present in a compound formation (Supplementary Table S6). The most abundant repeat motifs were mononucleotide (19,108; 43.38%) and complex (10,269; 23.31%), followed by trinucleotide (8109; 18.41%) and dinucleotide (5971; 13.56%) (Figure 7A). Pentanucleotide and hexanucleotide repeat motifs represented only 0.20% and 0.24% of the total SSRs, respectively. Taken together, 2812 types of nucleotide motif repeats were detected among 44,045 SSR loci. Among the trinucleotide SSRs, the most abundant repeat type was TGA/TCA (1058; 13.05%), followed by GAA/TTC (843; 10.40%), AGA/TCT (768; 9.47%), and ATC/GAT (631; 7.78%) (Figure 7B).

In order to evaluate the amplification efficiency of the newly developed SSR markers, a total of 25 markers based on the SSR-containing sequences were randomly selected for the validation and assessment of the polymorphism in 20 different cultivars. The motifs, primer information, and product size of the tested SSRs are listed in Supplementary Table S7. Among them, 14 pairs of primers were able to detect amplification products, of which 6 pairs of primers had insufficient polymorphism, which could not be used to distinguish different varieties, as less than five cultivars were detected, or the length of the products was not expected. The results of the nondenaturing polyacrylamide gels of the eight effective primers are shown in Figure 7C. The SSR molecular markers discovered in this work will aid in *A. roxburghii* breeding, germplasm resource identification, and the gene mining of critical agronomic features, all of which will assist in speeding up the directional breeding process.

## 4. Discussion

*A. roxburghii* is a rare, endangered herb. It is favored by people because it is rich in various medicinal and nutritional components, and its market demand has been increasing in recent years. In planting, the quality of *A. roxburghii* is mostly evaluated according to the polysaccharide content. Polysaccharide content refers to the amount of complex carbohydrates composed of long chains of monosaccharide units in a substance. The content and type of polysaccharides can influence the nutritional value, digestibility, and overall functionality of plant materials in food and other applications. Our results indicate that the polysaccharide level varies across different tissues and ages, which is consistent with those of previous studies [38]. As a biopolymer, polysaccharide is composed of monosaccharide units bound together by glycosidic linkages, and its biosynthesis is very complex [39]. Monosaccharides and disaccharides play different roles in plants. Monosaccharides, such as glucose, serve as a direct energy source and are building blocks for more complex carbohydrates. They are also involved in synthesizing vital molecules. In contrast, disaccharides, like sucrose, function primarily in energy storage and transport, providing energy when needed. Additionally, some disaccharides help plants withstand environmental stress. Our results indicate that the levels of various monosaccharides and disaccharides differ across the different tissues of *A. roxburghii*, which means that different tissues in have distinct metabolic activities and roles, leading to various concentrations of monosaccharides and disaccharides. For example, the leaves seem to have higher levels of monosaccharides, indicating active involvement in photosynthesis, while the roots store more disaccharides as energy reserves (Figure 1F).

The sequencing and de novo assembly of the transcriptome have become important strategies for the discovery of genes and molecular markers in non-model plants and other organisms [40,41,42,43]. While next-generation sequencing offers valuable resources for trait mapping and breeding, the absence of a complete genome sequence for *A. roxburghii* necessitates transcriptome analysis for effective gene annotation and further genomic studies. Here, we present transcriptomes of four tissues of a representative cultivar *A. roxburghii* from Guangdong (JXL28), China. The quality of the produced assembly was assessed with several statistics, revealing a highly contiguous (2277 N50) and complete (98.8% BUSCO scores for Eukaryote databases) transcriptome, with nearly 138,385 predicted unigenes. The number of unigenes assembled in *A. roxburghii* was lower than that reported for *A. formosanus* (173,513), but it was higher than that in *A. emeiensis* (78,381) [44] and even in *A. roxburghii* assembled using reads from PacBio Sequel II (72,666) [6]. All of the unigenes were successfully annotated in public databases (Nr, Nt, Swiss-Prot, COG, GO, and KEGG).

Understanding and utilizing the variability in agronomic traits among crop species are vital for sustainable agricultural practices. By promoting diversity and selecting crops with desirable traits, farmers and researchers can enhance the resilience, productivity, and sustainability of agroecosystems [45]. Significant progress has been made in identifying and understanding the mechanisms underlying morphological diversity by continuously updating sequencing technology in past research [46,47]. However, there are few reports on the molecular mechanisms behind the agronomic traits of *A. roxburghii*, leaving these mechanisms unclear.

Transcriptome sequencing enables accurate expression studies at the genomic level, especially for non-model organisms and those lacking genome sequence information. This study quantified variations in eight phenotypic traits and measured more than 10 repeats for each of the five *A. roxburghii* cultivars examined, as well as the chlorophyll and sucrose synthase content. Across the five cultivars evaluated here, the trends in both leaf number and plant height were consistent. This indicates that regardless of the cultivar, the changes in leaf number were paralleled by corresponding changes in plant height, suggesting a uniform growth pattern among the different cultivars. The chlorophyll content is closely related to the greenness of leaves. Chlorophyll, primarily chlorophyll a and chlorophyll b, is the main pigment in leaves. High chlorophyll content makes the leaves appear a deeper green because chlorophyll effectively absorbs red and blue light from the spectrum, reflecting green light. As shown in our results (Figure 3), the higher the chlorophyll content in the leaves, the more intense the green color (JXL15 and JXL28). Conversely, if the chlorophyll content is low, the leaves appear lighter green (JXL14) or even brown (JXL10 and JXL21).

Next, we conducted a comparative transcriptome analysis to determine the transcriptome changes among these five cultivars based on the de novo assembled JXL28 transcriptome. DEGs refer to genes whose expression levels significantly change under specific conditions or treatments compared to a control. DEGs can provide insights into the underlying mechanisms of a trait or phenotype, identifying DEGs is crucial in understanding various biological processes. A total of 30,688, 36,026, 36,959, and 29,892 DEGs were identified in four comparison groups: JXL10 vs. JXL28, JXL14 vs. JXL28, JXL15 vs. JXL28, and JXL21 vs. JXL28. Additionally, a total of 9033 DEGs were identified in all comparisons.

To further analyze these DEGs, we employed *K*-means clustering, an effective method for classifying global samples and enhancing the accuracy of co-expression analyses [48]. Here, we obtained 10 clusters based on gene expression, among which five clusters represented genes specifically highly expressed in each cultivar. The WGCNA has been widely applied to effectively investigate the relation between genome and phenotype [49,50,51]. We built a gene co-expression network to more precisely mine the core genes linked to chlorophyll content and sucrose synthase activity. A gene hierarchical clustering dendrogram was constructed via gene correlation, and a total of nine gene modules were identified. Among them, the red module had the highest negative correlation (R = −0.76) with chlorophyll content, while the yellow module had the highest positive correlation (R = 0.55). By calculating the GS and MM values, we identified the most central genes in the modules, of which eight genes were selected for assessment using RT-qPCR. Additionally, the RT-qPCR experiments showed that, for most genes, the expression levels in the leaves of *A. roxburghii* were higher than those in the stems. We speculated that the polysaccharide content affects the efficiency of RNA extraction, which is consistent with the higher polysaccharide content being higher in the stems than in the leaves. Our results provide a reference for the molecular regulatory network and key genes in the development of leaves and contribute to molecular breeding of morphological traits in *A. roxburghii*.

Utilizing NGS technologies, transcriptome sequencing provides a rapid and cost-effective method for discovering SNPs and SSRs within coding regions, which are more likely associated with diverse biological functions. SSR loci, or Simple Sequence Repeat loci, refer to regions in the genome containing short, repeating sequences of one to six base pairs, known as microsatellites. These loci are highly polymorphic, meaning they exhibit significant variation in the number of repeat units among individuals within a population. Expressed sequence tags (ESTs) serve as a valuable asset in the identification of new genes and contribute to the genome annotation process in many crops [13,52,53]. Moreover, EST-SSRs exhibit a greater level of transferability to closely related species than genomic SSR markers [54,55]. It is essential to expedite the advancement of crop improvement initiatives by employing molecular markers, particularly in the context of molecular breeding, as a more suitable alternative to conventional breeding. In this study, a total of 44,045 SSR loci with a frequency of 25.25% (34,946/13,8385) were identified from 34,946 unigenes of the *A. roxburghii* transcriptome under the MISA screening conditions. Compared with other Orchidaceae plants, the occurrence frequency of SSR loci was higher than that of *A. emeiensis* (9.88%) [44], *Gastrodia elata* Blume (5.79%) [56], Denphal-type *Dendrobium* (7.11%) [57], and *Cymbidium ensifolium* (7.82%) [58], but lower than that of *Phalaenopsis aphrodite* (33.33%) [59]. It has been reported that the use of various bioinformatics software tools, search criteria, and database sizes for identifying microsatellites can lead to variations in SSR locus frequencies [60]. In the simple screening of 20 cultivars, eight primer pairs were effective polymorphic. These should be used to test for polymorphism in more individuals. SSR locus analyses in transcriptome and molecular marker development are valuable resources for further studies on marker-assisted selection in *A. roxburghii* breeding. Overall, these markers will aid in investigating the genetic structure of populations and help us understand the molecular synthesis mechanisms of the medicinal components in *A. roxburghii*.

## 5. Conclusions

In this study, we report a high-quality de novo assembled and characterized transcriptome for *Anoectochilus roxburghii* that is useful for functional genomics and molecular genetic studies. The agronomic characteristics of JXL10, JXL14, JXL15, JXL21, and JXL28 (five cultivars with obvious phenotypic differences) were investigated. In addition, through the analysis and mining of comparative transcriptomes, species-specific and possible functional genes were identified to provide information for understanding the morphological development strategies of five typical *A. roxburghii* cultivars. Moreover, the transcriptome-derived SSR markers were examined among 20 populations. The EST-SSR markers examined in this study are informative for future population genetic studies of *A. roxburghii*. This article provides an important basis for future studies on genetics research of *A. roxburghii* and further promotes biotechnological applications of *A. roxburghii*.

## Data Availability

The datasets generated and analyzed during the current study are available in the BIG Submission (BIG Sub, https://ngdc.cncb.ac.cn/gsub/ (accessed on 22 July 2022)) of China National Center for Bioinformation (CNCB) under the project number PRJCA028087. The other supporting data are included as Supplementary Materials.

## Supplementary Materials

The following supporting information can be downloaded at: https://www.mdpi.com/article/10.3390/plants13233262/s1, Figure S1: Cluster heatmap of each differential group. The diagram presents the result of a two-way hierarchical clustering of all the DEGs and comparison combinations. The *X*-axis represents the sample name and hierarchical clustering results, and the *Y*-axis represents the differentially expressed genes and hierarchical clustering results. Red represents high expression, and green represents low expression; Figure S2: A heatmap of all unigenes and a hierarchical cluster analysis of all five cultivars; Figure S3: A correlation analysis of all five cultivars based on the expression of all unigenes; Figure S4: The identification of DEGs for four cultivars compared with JXL28. The *X*-axis represents log2FC, and the *Y*-axis represents -log10 (*p* value). The green dots represent the number of down-regulated unigenes. The red dots represent the number of up-regulated unigenes; Figure S5: The results of all ten subclasses from the *K*-means clustering analysis; Figure S6: A GO enrichment analysis of the five main clusters; Figure S7: The centrality of key genes for chlorophyll a (A), chlorophyll b (B), total chlorophyll (C), and sucrose synthase (D) content was determined based on module membership (MM) and gene significance (GS). The *X*-axis MM represents the correlation between genes and modules, and the *Y*-axis GS represents the correlation between genes and traits; Figure S8: KEGG enrichment analysis of genes in red (A) and black (B) module. Table S1. Sequencing summary of all samples. Table S2. Statistical summary of the de novo assembly of JXL28. Table S3. Level detection of 13 sugar components in different tissues. Table S4. Annotation results of unigene functions to different databases. Table S5. Details of the primers designed and screened for amplification in Anoectochilus roxburghii cultivars. Table S6. Detailed information of identifed SSRs. Table S7. Validation of SSR motifs and polymorphisms for 25 primer pairs.
