# Supplementary material for: De Novo Transcriptome Assembly of Anoectochilus roxburghii for Morphological Diversity Assessment and Potential Marker Development
Source: Plants (Basel). 2024 Nov 21;13(23):3262. doi: 10.3390/plants13233262 (PMC11644659; doi:10.3390/plants13233262)
Supplement: Supplementary file 1 [file plants-13-03262-s001.zip › Figure S1.pdf]

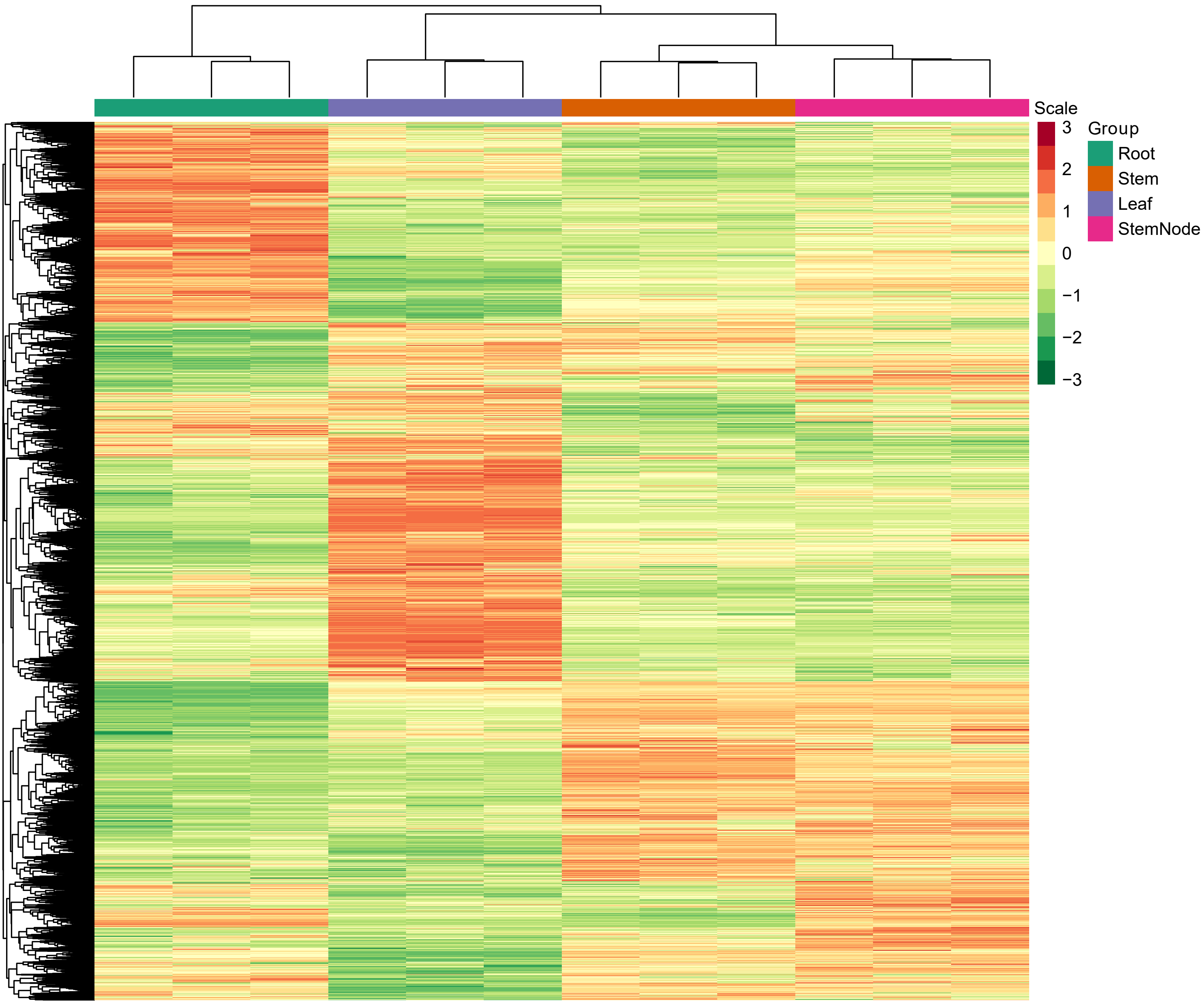

Figure S1: Cluster heatmap of each differential group. The diagram presents the result of a two-way hierarchical clustering of all the DEGs and comparison combinations. The X-axis represents the sample name and hierarchical clustering results, and the Y-axis represents the differentially expressed genes and hierarchical clustering results. Red represents high expression, and green represents low expression
