# Supplementary material for: De Novo Transcriptome Assembly of Anoectochilus roxburghii for Morphological Diversity Assessment and Potential Marker Development
Source: Plants (Basel). 2024 Nov 21;13(23):3262. doi: 10.3390/plants13233262 (PMC11644659; doi:10.3390/plants13233262)
Supplement: Supplementary file 1 [file plants-13-03262-s001.zip › Figure S4.pdf]

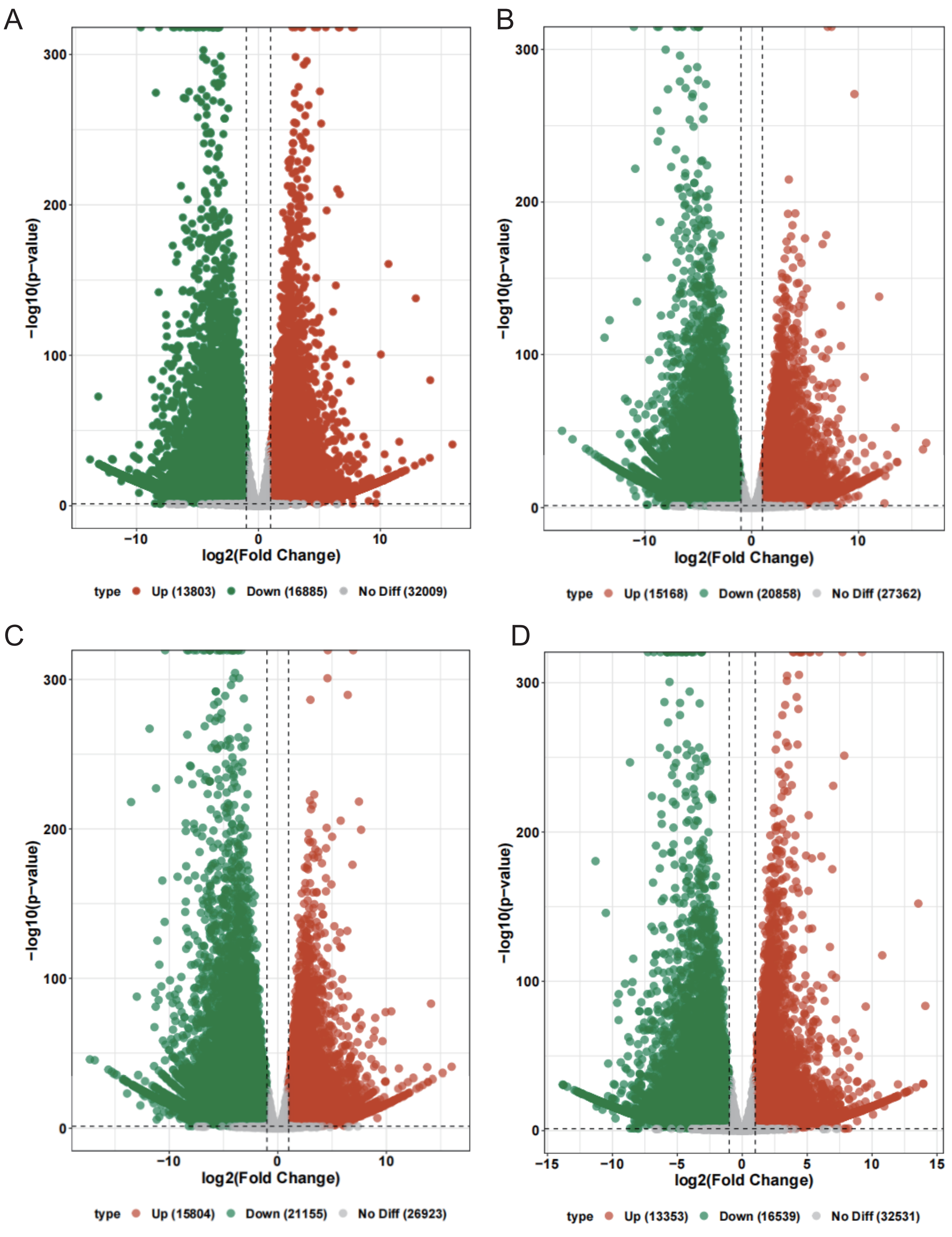

Figure S4: The identification of DEGs for four cultivars compared with JXL28. The X-axis represents log$_2$FC, and the Y-axis represents -log10 (*p* value). The green dots represent the number of down-regulated unigenes. The red dots represent the number of up-regulated unigenes.
