# Supplementary material for: De Novo Transcriptome Assembly of Anoectochilus roxburghii for Morphological Diversity Assessment and Potential Marker Development
Source: Plants (Basel). 2024 Nov 21;13(23):3262. doi: 10.3390/plants13233262 (PMC11644659; doi:10.3390/plants13233262)
Supplement: Supplementary file 1 [file plants-13-03262-s001.zip › Figure S7.pdf]

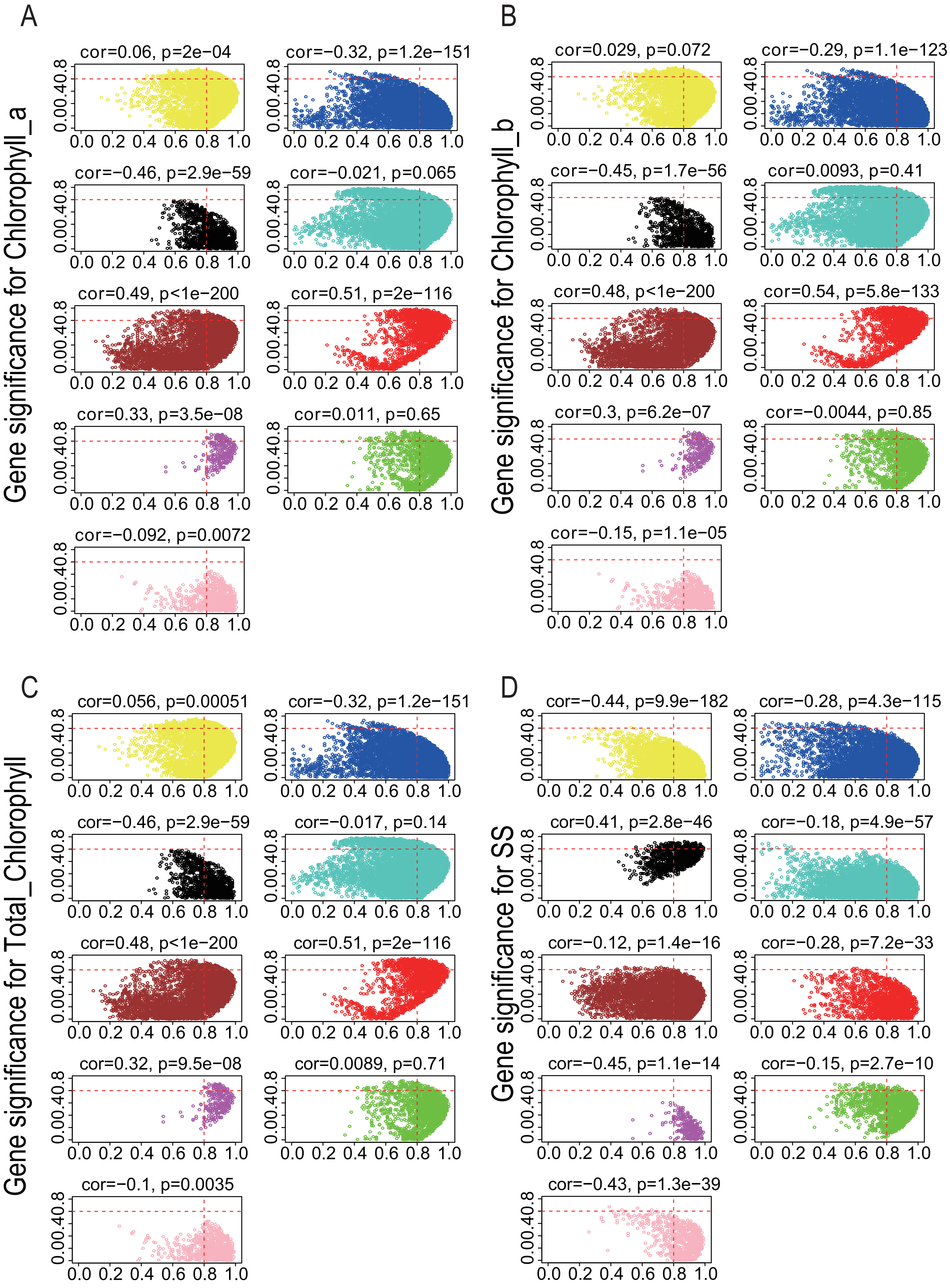
A
Gene significance for Chlorophyll_a
cor=0.06, p=2e−04
cor=−0.32, p=1.2e−151
cor=−0.46, p=2.9e−59
cor=−0.021, p=0.065
cor=0.49, p<1e−200
cor=0.51, p=2e−116
cor=0.33, p=3.5e−08
cor=0.011, p=0.65
cor=−0.092, p=0.0072
B
Gene significance for Chlorophyll_b
cor=0.029, p=0.072
cor=−0.29, p=1.1e−123
cor=−0.45, p=1.7e−56
cor=0.0093, p=0.41
cor=0.48, p<1e−200
cor=0.54, p=5.8e−133
cor=0.3, p=6.2e−07
cor=−0.0044, p=0.85
cor=−0.15, p=1.1e−05
C
Gene significance for Total_Chlorophyll
cor=0.056, p=0.00051
cor=−0.32, p=1.2e−151
cor=−0.46, p=2.9e−59
cor=−0.017, p=0.14
cor=0.48, p<1e−200
cor=0.51, p=2e−116
cor=0.32, p=9.5e−08
cor=0.0089, p=0.71
cor=−0.1, p=0.0035
D
Gene significance for SS
cor=−0.44, p=9.9e−182
cor=−0.28, p=4.3e−115
cor=0.41, p=2.8e−46
cor=−0.18, p=4.9e−57
cor=−0.12, p=1.4e−16
cor=−0.28, p=7.2e−33
cor=−0.45, p=1.1e−14
cor=−0.15, p=2.7e−10
cor=−0.43, p=1.3e−39

Figure S7: The centrality of key genes for chlorophyll a (A), chlorophyll b (B), total chlorophyll (C), and sucrose synthase (D) content was determined based on module membership (MM) and gene significance (GS). The X-axis MM represents the correlation between genes and modules, and the Y-axis GS represents the correlation between genes and traits.
