# Supplementary figures and images for: De Novo Transcriptome Assembly of Anoectochilus roxburghii for Morphological Diversity Assessment and Potential Marker Development
Source: Plants (Basel). 2024 Nov 21;13(23):3262. doi: 10.3390/plants13233262 (PMC11644659; doi:10.3390/plants13233262)

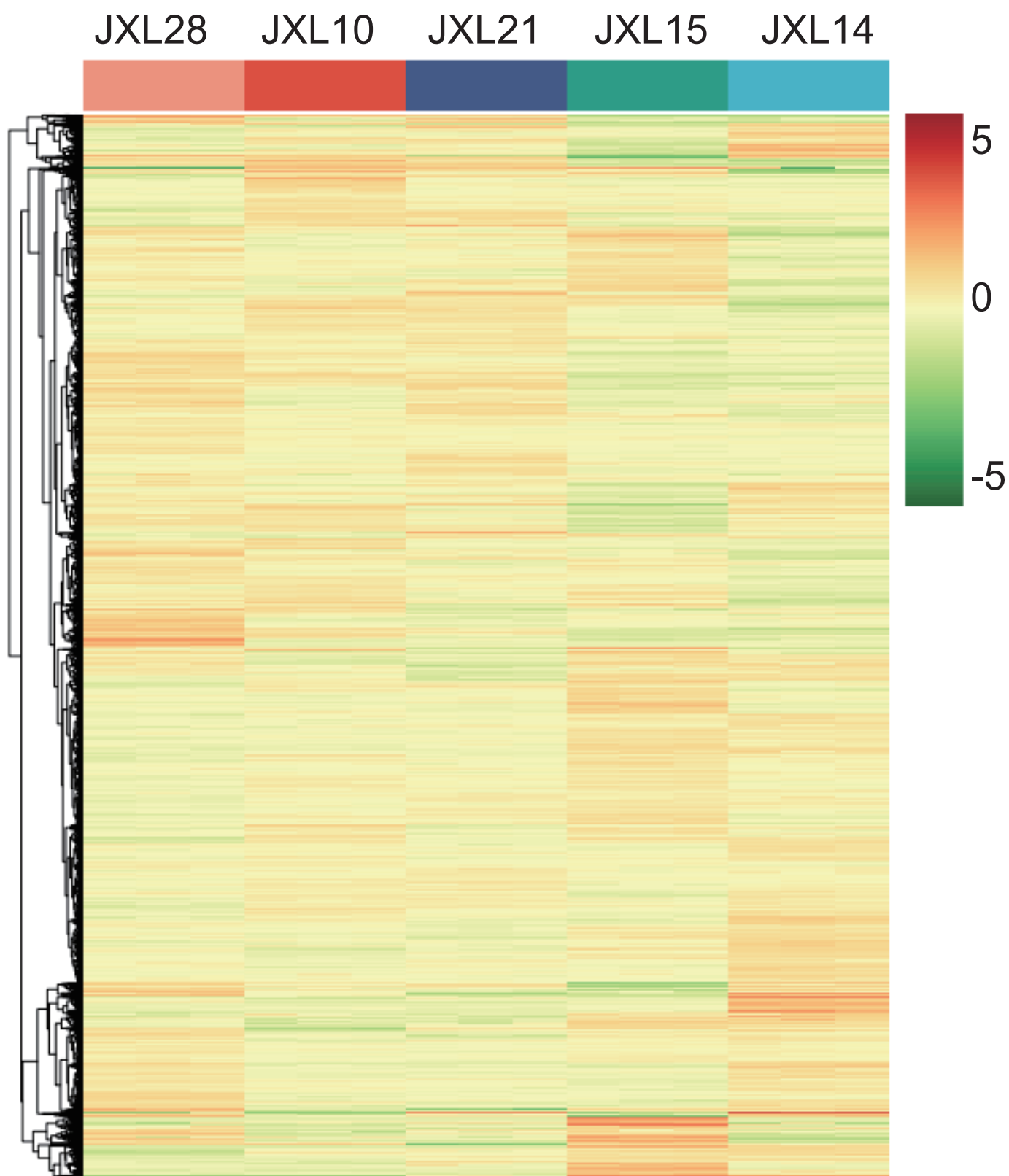

Figure S2: A heatmap of all unigenes and a hierarchical cluster analysis of all five cultivars

Supplement: Supplementary file 1 [file plants-13-03262-s001.zip › Figure S2.pdf]

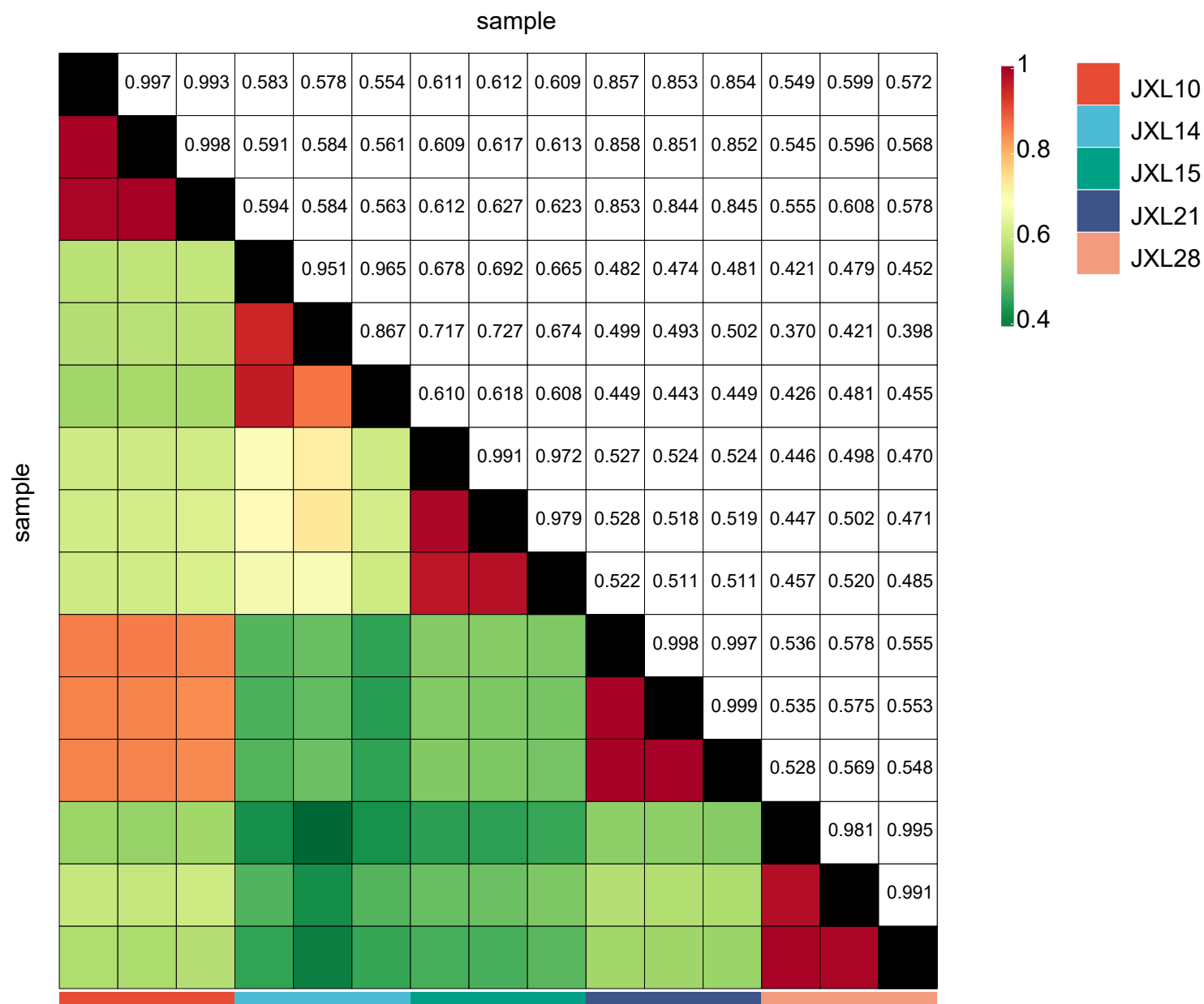

Figure S3: A correlation analysis of all five cultivars based on the expression of all unigenes

Supplement: Supplementary file 1 [file plants-13-03262-s001.zip › Figure S3.pdf]

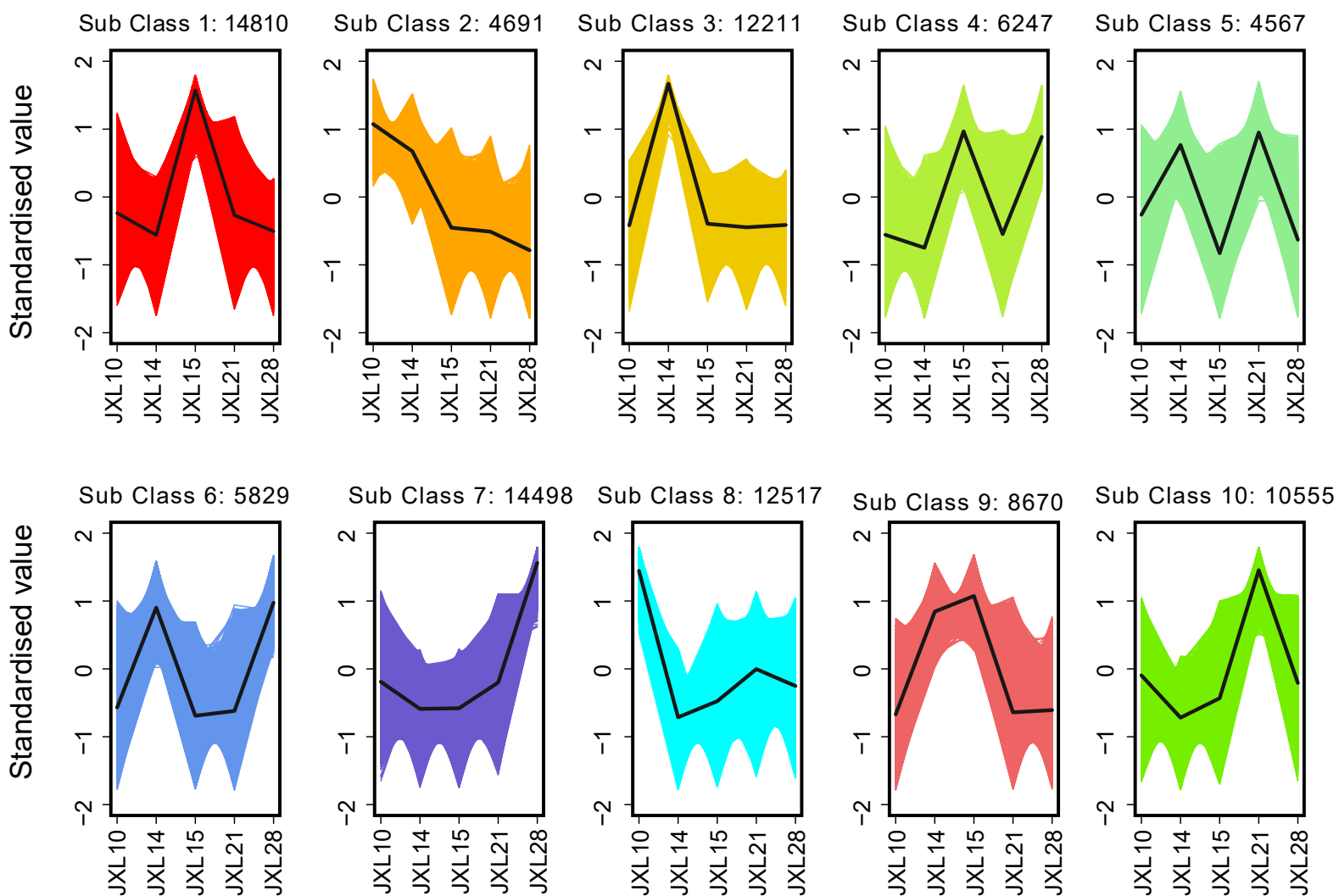

Figure S5: The results of all ten subclasses from the K-means clustering analysis.

Supplement: Supplementary file 1 [file plants-13-03262-s001.zip › Figure S5.pdf]

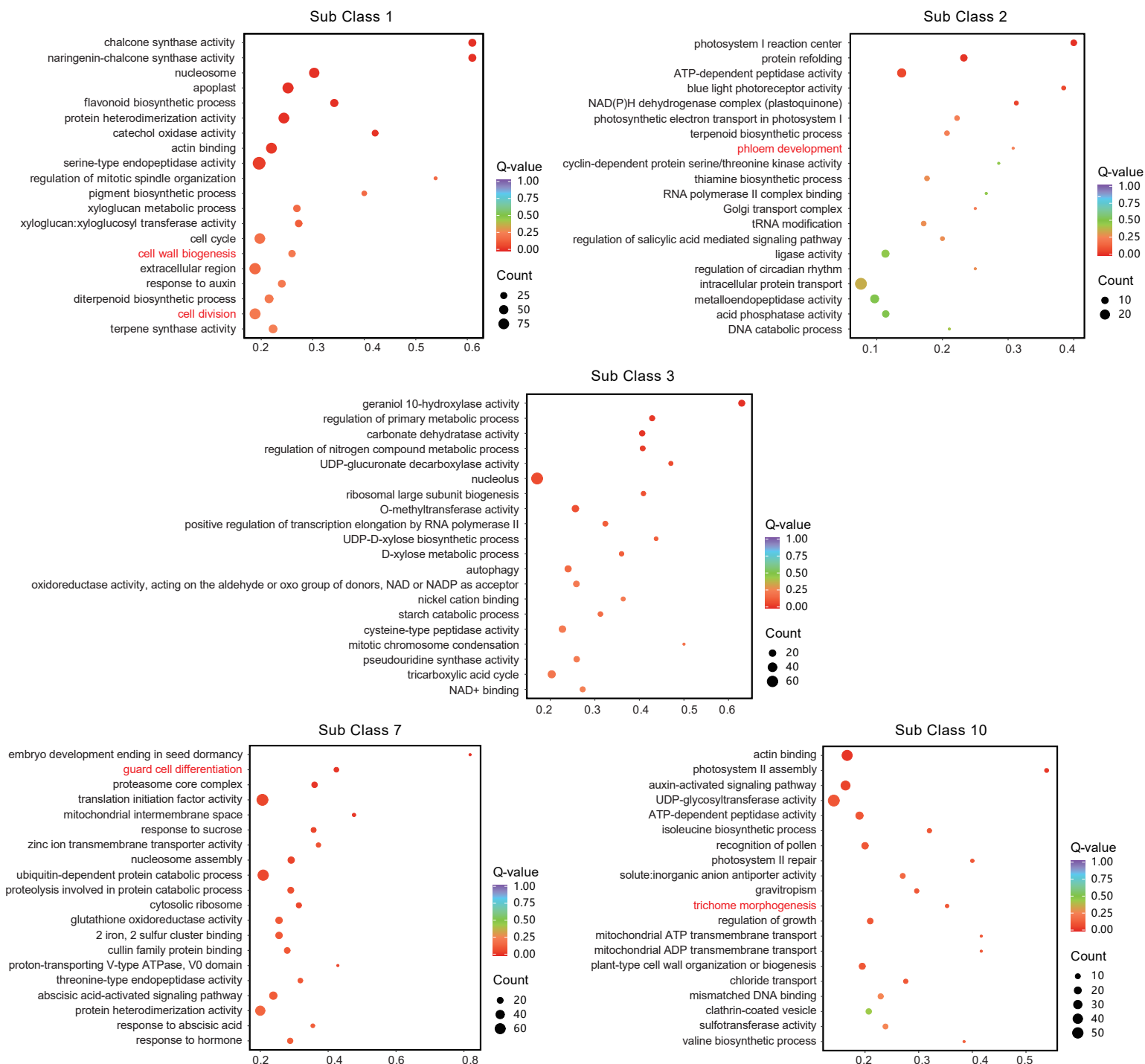

Figure S6: A GO enrichment analysis of the five main clusters

Supplement: Supplementary file 1 [file plants-13-03262-s001.zip › Figure S6.pdf]

A

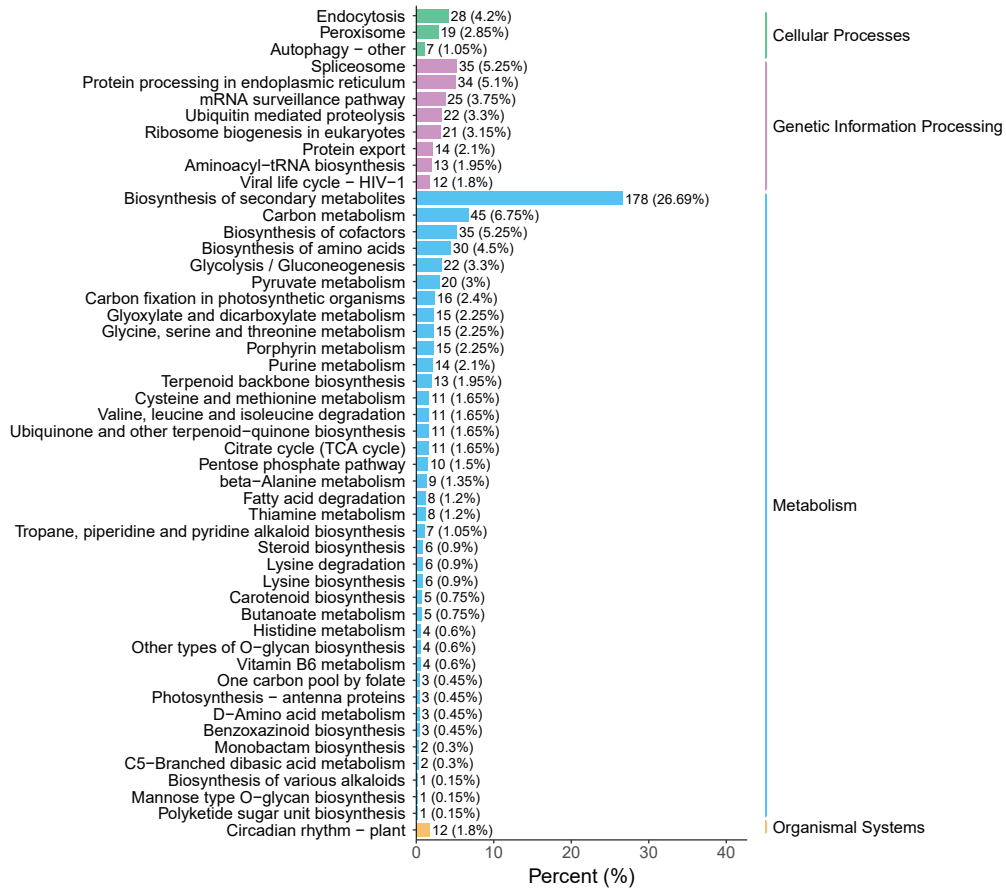

B

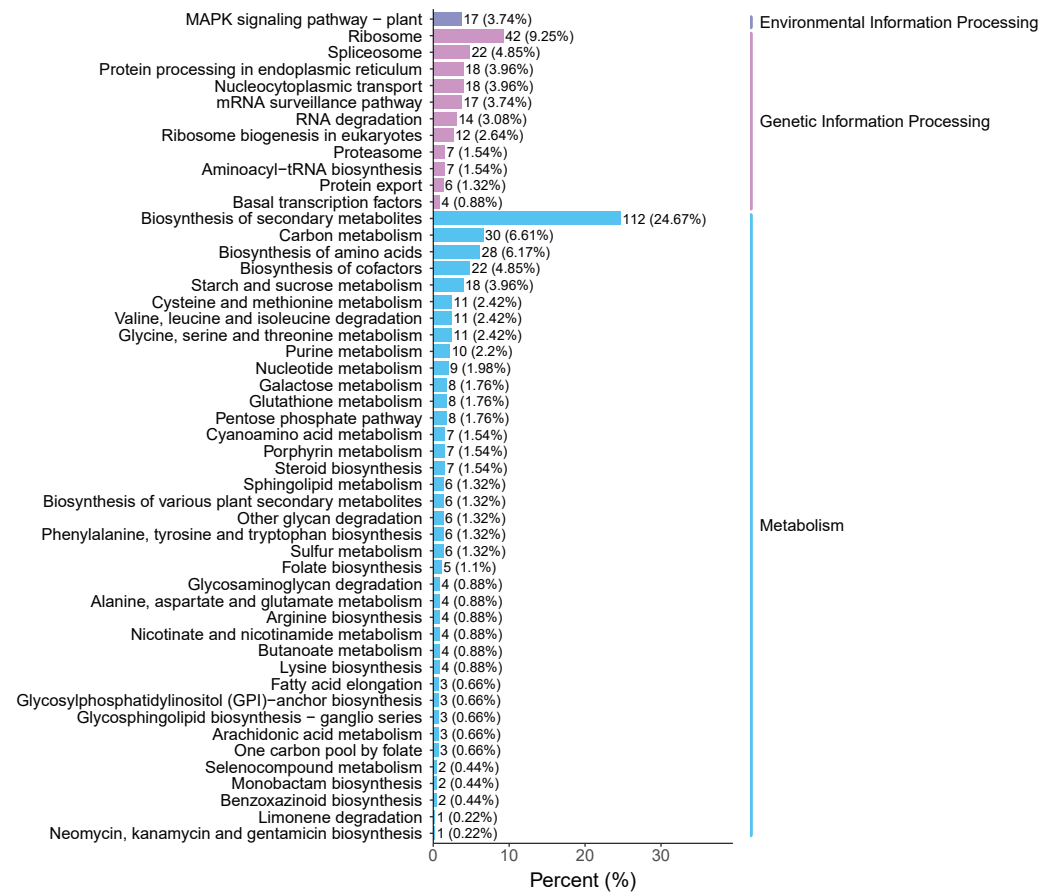

Figure S8: KEGG enrichment analysis of genes in red (A) and black (B) module

Supplement: Supplementary file 1 [file plants-13-03262-s001.zip › Figure S8.pdf]
